# Development and Validation of an Interpretable Machine Learning Model Based on Routine Blood Biomarkers: For Predicting Age-Related Hearing Loss

**DOI:** 10.3390/diagnostics16132025

**Published:** 2026-06-29

**Authors:** Dan He, Yiting Liu, Jing Ke, Xu Jiang, Haiyu Ma, Ya Shi, Wei Yuan

**Affiliations:** 1 Chongqing Medical University, Chongqing 400016, China; 2 Department of Otorhinolaryngology-Head and Neck Surgery, Chongqing General Hospital, Chongqing University, Chongqing 400016, China; 3 School of Medicine, Chongqing University, Chongqing 400016, China

**Keywords:** age-related hearing loss, machine learning, blood biomarkers, interpretability, predictive model

## Abstract

**Background/Objectives:** Age-related hearing loss (ARHL) is a common sensory impairment in the elderly, and its early prediction and intervention are crucial for improving the quality of life in older adults. This study aims to develop and validate an interpretable machine learning model based on routine blood biomarkers to predict the risk of ARHL occurrence. **Methods:** A total of 542 participants were selected from the National Health and Nutrition Examination Survey (NHANES) database, including 271 ARHL patients and 271 healthy controls. The samples were randomly divided into a training set (50%) and two independent internal validation sets (25% each). Through systematic comparison of 113 machine learning algorithm combinations, the optimal predictive model (glmBoost+Stepglm[forward]) was constructed, and the SHAP method was employed for feature interpretation. To evaluate the model’s generalization ability, external validation was further performed using a cohort of 92 cases from Chongqing People’s Hospital. Additionally, an openly accessible interactive prediction web page was developed based on the R Shiny framework, supporting real-time clinical risk assessment and visual interpretation. **Results:** The model achieved an AUC of 0.948 in the training set, with AUCs of 0.893 and 0.945 in two internal validation sets, respectively, and an overall accuracy rate of 86.3%. In the external validation cohort (albeit with a limited sample size of 92 from a single center), the model maintained good performance with an AUC of 0.839 (95% CI: 0.750–0.918) and an accuracy of 77.2%. The model identified nine key predictive features, with the top three being glycated hemoglobin (HbA1c), mean corpuscular volume (MCV), and blood glucose according to SHAP interpretability analysis. **Conclusions:** This study successfully developed and validated an interpretable machine learning model based on routine blood biomarkers for community-based risk stratification of age-related hearing loss. The model demonstrated robust performance in internal and external validations, including an age-matched elderly subgroup. An interactive web tool was developed to facilitate real-time risk assessment. While the model is intended as a prescreening tool for large-scale populations rather than a diagnostic test for age-matched individuals, it provides a novel approach for early identification of individuals at higher risk of ARHL and offers insights into its systemic pathogenesis.

## 1. Introduction

Age-related hearing loss (ARHL) is increasingly recognized as a significant public health issue and ranks as the third leading cause of chronic disability among the elderly [1,2]. ARHL is characterized by a gradual decline in hearing, particularly at high frequencies, which often leads to communication difficulties, social isolation, and reduced quality of life in the elderly [3,4,5]. In addition to hearing impairment, ARHL is also associated with various adverse outcomes, such as emotional distress, cognitive decline, and an increased risk of dementia [6,7]. Currently, the clinical diagnosis of ARHL primarily relies on audiological assessments conducted after significant hearing loss has occurred, lacking effective early warning and risk prediction methods. This results in delayed intervention and missed critical windows for prevention and treatment. Existing interventions such as hearing aids and cochlear implants exhibit substantial individual variability in effectiveness, with a notable absence of objective biomarkers and evaluation systems capable of accurately predicting intervention responses and guiding personalized rehabilitation plans. Therefore, there is an urgent need to develop predictive and risk assessment tools that can identify high-risk individuals during early or even preclinical stages, enabling proactive intervention and delaying disease progression. This holds significant social implications and promising application prospects.

In recent years, artificial intelligence and machine learning technologies have achieved groundbreaking progress in the field of medical prediction, providing a new paradigm for early identification of complex diseases. By uncovering high-dimensional, nonlinear data relationships, machine learning models can integrate multiple risk factors to achieve more precise risk stratification [8,9]. However, the application of this technology in hearing loss research remains in its infancy. Existing studies predominantly focus on imaging or genomic data [10], with a primary emphasis on noise-induced hearing impairment [11,12,13,14]. Models constructed based on routinely available clinical indicators remain scarce, particularly for presbycusis, for which no studies currently exist. More importantly, many machine learning models function as “black boxes,” lacking interpretability in their predictive logic, which significantly hinders their credibility and clinical translation in decision-making processes.

On the other hand, substantial evidence indicates that ARHL is not merely an isolated aging process of the auditory organ, but rather intricately intertwined with systemic aging and multiple comorbid conditions [15,16]. Conventional blood biomarkers—such as glycated hemoglobin (HbA1c) reflecting glucose metabolism status [17], fasting blood glucose [18], and various lipid parameters indicative of lipid metabolism [19]—have been demonstrated to be closely associated with hearing loss. However, the predictive value of these biomarkers for ARHL has not yet been systematically integrated and evaluated; moreover, how they interact and collectively influence hearing loss risk remains poorly understood due to a lack of in-depth interpretation based on big data models.

### Novelties and Contributions

This study offers several novel contributions. It is the first machine-learning model for ARHL prediction using exclusively routine blood biomarkers, with systematic comparison of 113 algorithms across 12 frameworks. It provides comprehensive SHAP-based interpretability to reveal feature contributions and interactions. It also includes external validation in an independent Chinese clinical cohort and offers a publicly accessible interactive web tool for real-time risk assessment. To address the aforementioned challenges and bridge the existing research gaps, this study aims to develop and validate an interpretable machine learning model based on routine blood biomarkers using a nationally representative large-scale public health database—the National Health and Nutrition Examination Survey (NHANES). The specific objectives of this study are: first, to construct an ensemble model capable of accurately predicting the risk of ARHL in elderly individuals; second, to rigorously validate the model’s generalization performance in independent samples; and third, to employ advanced model interpretability techniques (such as SHAP) to thoroughly analyze the contribution direction and patterns of various blood biomarkers to the prediction outcomes, thereby revealing their potential complex relationships with ARHL. We anticipate that this model will not only serve as an efficient early screening tool, but also provide novel data-driven insights into the multifactorial and heterogeneous pathogenesis of ARHL, thereby laying the foundation for developing targeted prevention and treatment strategies in the future.

## 2. Methods

### 2.1. Study Design and Data Sources

This study is a retrospective cohort analysis aimed at constructing and validating a predictive model for age-related hearing loss (ARHL) using large-scale public health data. The data were sourced from the National Health and Nutrition Examination Survey (NHANES) database (https://www.cdc.gov/nchs/nhanes/index.html, accessed on 9 December 2024). NHANES is a nationally representative cross-sectional survey conducted by the Centers for Disease Control and Prevention (CDC), employing a multistage probability sampling method to systematically collect multidimensional health information, including audiometry, physical examinations, and laboratory tests, providing a high-quality data foundation for this study.

### 2.2. Study Population Screening

**Figure 1:**
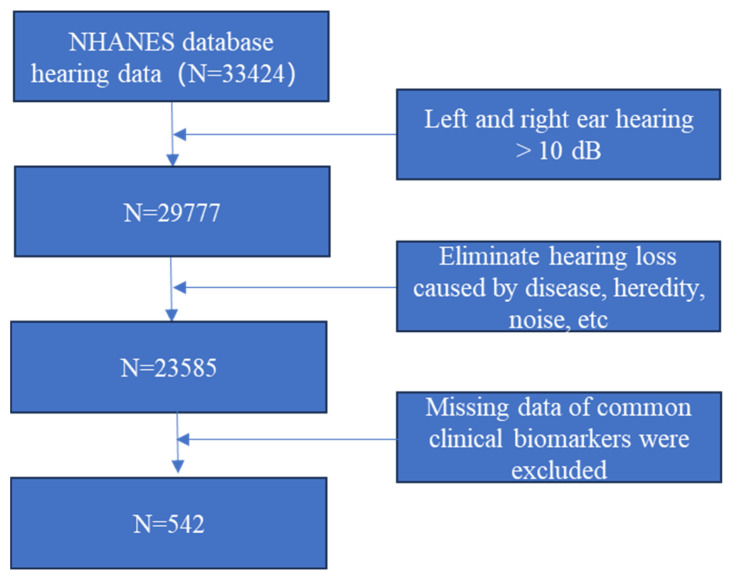
Flowchart of patient inclusion.

According to the research objectives, we conducted stepwise participant screening following these procedures (see Figure 1 for the flowchart): First, we extracted all individuals who completed pure-tone audiometry (500–8000 Hz) from NHANES, totaling 33,424 participants. To exclude asymmetric hearing loss, we retained only those with interaural differences in pure-tone average thresholds (500, 1000, 2000, 4000 Hz) < 10 dB HL, leaving 29,777 participants. Based on questionnaire and physical examination data, we further excluded individuals with definite non-age-related etiologies, including: active middle ear diseases or history of ear surgery, long-term high-intensity noise exposure, family history of hereditary hearing loss, and history of ototoxic medication use. This step resulted in 23,585 remaining participants. After excluding participants with missing key blood biomarker data, a total of 542 participants were ultimately included in the analysis. It should be noted that the control group was selected to include individuals with normal hearing across all available age ranges in the NHANES database, as the number of elderly participants with normal hearing was insufficient for strict age-matching in the training set. To mitigate this inherent imbalance, we performed an age-matched external validation using the independent Chongqing cohort (detailed in the Results section), which confirmed that the model maintains high discriminative ability even when chronological age is strictly balanced between groups.

### 2.3. Definition, Classification and Dataset Partitioning of Hearing Loss

ARHL group: Age ≥60 years [20,21] with bilateral pure-tone average (PTA) ≥30 dB HL [22]. Control group: Bilateral PTA < 30 dB HL. A total of 271 cases were included in each group. To evaluate model performance, the total sample (*N* = 542) was randomly divided into: Training set: 50% (*N* = 271), used for model construction and tuning. Validation set 1 and validation set 2: Each accounting for half of the remaining 50% (approximately 135–137 cases each), used to test the model’s generalization capability.

### 2.4. Feature Extraction

Routine blood biomarkers: including glycated hemoglobin (HbA1c), fasting blood glucose, uric acid, lipid profile (total cholesterol, HDL-C, LDL-C, triglycerides), albumin, total protein, white blood cell count, lymphocyte count, neutrophil count, monocyte count, platelet count, mean corpuscular volume (MCV), red cell distribution width, etc. All blood indicators were measured according to the standardized laboratory procedures of NHANES.

### 2.5. Machine Learning Model Construction

Data preprocessing: Perform Z-score standardization on the training set features and apply the same transformation to the validation set. Model comparison and selection: Systematically compare 113 algorithms covering 12 categories of machine learning frameworks (such as logistic regression, support vector machines, random forests, gradient boosting machines, neural networks, etc.). Employ 10-fold cross-validation, using AUC as the primary metric combined with accuracy for evaluation. Final model: The glmBoost+Stepglm[forward] model demonstrated optimal performance, further refined through hyperparameter tuning via grid search and Bayesian optimization.

### 2.6. External Validation Cohort

To assess the model’s generalizability and clinical applicability, we additionally collected an independent external validation cohort from Chongqing People’s Hospital. Each patient and volunteer provided written informed consent before sampling. This cohort included patients who visited between October 2025 and December 2025 and completed pure-tone audiometry and relevant blood tests. All patients met the same exclusion criteria as the NHANES cohort (e.g., active middle ear disease, history of noise exposure, etc.). Blood biomarker testing followed standardized hospital laboratory procedures. This cohort was used to independently evaluate the predictive performance of the constructed machine learning model in real-world clinical settings.

### 2.7. Model Validation and Interpretability Analysis

Performance evaluation: The following metrics were calculated on two independent validation sets. Discriminative ability: AUC. Classification performance: accuracy, sensitivity, specificity, positive predictive value, and negative predictive value. Interpretability Analysis: Employing the SHAP (SHapley Additive exPlanations) framework. Global interpretation: Feature importance and impact direction are visualized through SHAP summary plots. Local interpretation: SHAP dependence plots, decision force plots, and waterfall plots are utilized to reveal the influence of key features on individual prediction outcomes and the interaction relationships between features.

### 2.8. Development of Interactive Prediction Web Interface

To facilitate convenient model usage and result visualization, we developed a publicly accessible interactive web-based tool using the R Shiny framework (Web-based Interactive Tool). This web application enables users to input values for nine key blood biomarkers via their browser to obtain real-time personalized risk prediction for age-related hearing loss, with dynamic visualization of SHAP explanation graphs (such as decision plots). The frontend employs responsive design to ensure compatibility across different devices, while the backend integrates a pre-trained glmBoost+Stepglm[forward] model and standardized processing pipeline to maintain consistency with the research prediction logic. Deployed on the ShinyApps.io platform (https://kj888.shinyapps.io/glmBoost-Stepglm/, accessed on 9 December 2024), the application requires no local installation and is suitable for rapid clinical assessment and academic demonstrations.

### 2.9. Statistical Analysis

All analyses were performed using R (version 4.3.0) and Python (version 3.9). Continuous variables conforming to normal distribution were presented as mean ± standard deviation, with between-group comparisons conducted using independent samples *t*-test; otherwise, they were expressed as median (interquartile range) and analyzed using Mann–Whitney U test. Spearman correlation coefficient was employed to assess the correlation between features and hearing thresholds. All tests were two-tailed, with *p* < 0.05 considered statistically significant. The R scripts and preprocessing pipeline used in this study are available in the Supplementary Materials.

## 3. Results

### 3.1. Study Participant Screening Process

As shown in Figure 1, through multi-level screening, a total of 542 participants were ultimately enrolled, with 271 cases each in the ARHL group and control group.

### 3.2. Comparison of Baseline Characteristics

As shown in Table 1, a systematic comparison was conducted between the presbycusis group and the healthy control group regarding demographic, audiological, and blood biochemical indicators. In addition to the highly significant differences in age and hearing thresholds, multiple metabolic-related indicators (such as HbA1c, blood glucose, uric acid, and triglycerides) and inflammatory-immune indicators (such as lymphocyte and monocyte ratios) also exhibited significant intergroup differences. These findings provide crucial clinical evidence for the subsequent construction of a multifactorial predictive model.

### 3.3. Correlation Analysis of Characteristics

**Figure 2:**
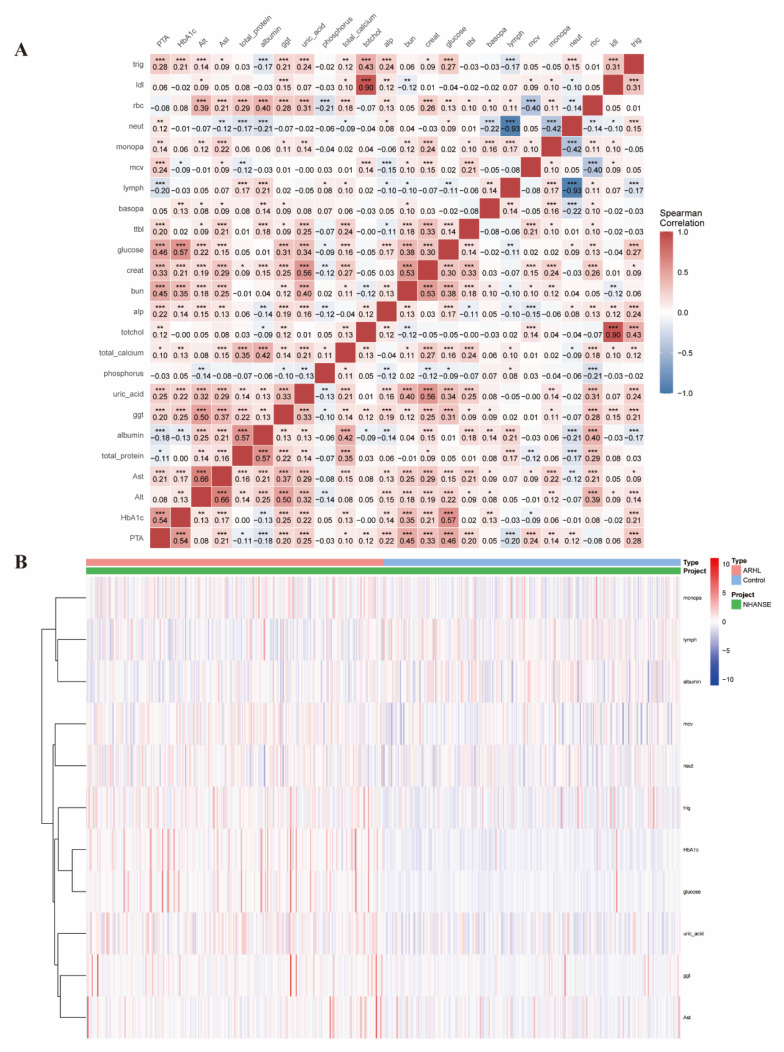
Correlation analysis between blood biomarkers and hearing loss with differential indicator screening. (**A**) Correlation analysis between blood biomarkers and PTA. * represents *p* < 0.05, ** represents *p* < 0.01, *** represents *p* < 0.001. (**B**) Differential expression analysis of blood biomarkers between ARHL and control groups.

Figure 2 displays the correlation between various blood biomarkers and pure-tone average (PTA) thresholds, along with the screening results. Figure 2A presents the correlation analysis between all indicators and PTA, revealing that certain metabolic and inflammation-related indicators (such as HbA1c and lymphocyte percentage) showed significant correlations with hearing thresholds. Through further intergroup comparison, Figure 2B highlights the blood biomarkers that exhibited statistically significant differences (*p* < 0.05) between the ARHL group and the control group. These indicators provided crucial evidence for subsequent modeling.

### 3.4. Performance of Machine Learning Models

**Figure 3:**
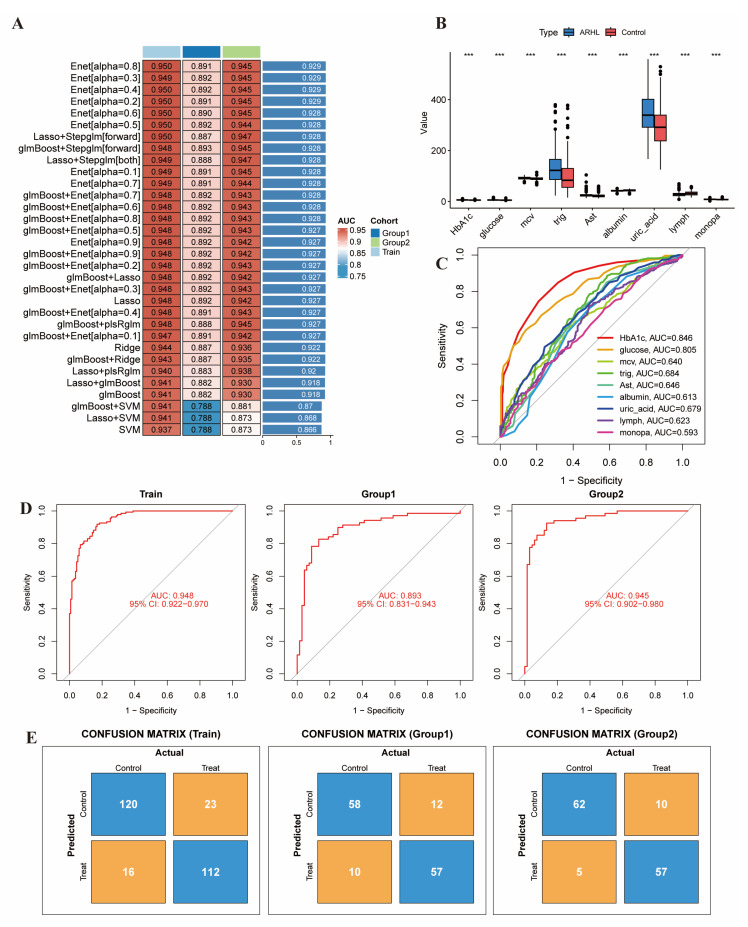
Construction, performance evaluation and key predictive features of machine learning models. (**A**) Comparison of AUC among different machine learning combinations. (**B**) Expression patterns of 9 indicators used for model construction across different groups. *** represents *p* < 0.001. (**C**) AUC comparison chart of models constructed with individual indicators. (**D**) ROC curves of glmBoost+Stepglm[forward] in different datasets. (**E**) Results of confusion matrices in different datasets.

This study systematically screened 113 algorithm combinations encompassing 12 categories of machine learning frameworks (such as logistic regression, support vector machines, random forests, gradient boosting machines, neural networks, etc.) to construct a presbycusis prediction model (Figure 3A). The glmBoost+Stepglm[forward] model demonstrated optimal performance among them. It is relatively high across data sets, with Group 1 being the highest. So we decided that this model was the most stable, and we chose this as the final model. This model incorporated the following 9 features: HbA1c, glucose, uric_acid, trig, albumin, Ast, mcv, lymph. The nine clinical features on which the model relies all showed significant differences between presbycusis patients and controls (Figure 3B), providing an interpretable biological basis for the model. Notably, the predictive performance of any single indicator (highest AUC = 0.846 for HbA1c) was substantially lower than that of this integrated model (Figure 3C), emphasizing the necessity of incorporating multidimensional information for achieving precise prediction. The model demonstrated near-perfect discriminative ability on the training set (AUC = 0.948) and maintained excellent generalization performance in two independent validation sets (Group1 and Group2), with AUC values of 0.893 and 0.945, respectively (Figure 3D). The confusion matrix further revealed (Figure 3E) that the model achieved accuracies of 83.9% (115/137) in Group1, 88.8% (119/134) in Group2, and 85.6% (232/271) in the training set. The overall accuracy in the validation sets reached 86.3% (234/271), reflecting high and balanced sensitivity and specificity. In the validation set, sensitivity was 83.8% (114/136), precision was 88.4% (114/129), F1 score was 86.1%, and specificity was 88.9% (120/135).

### 3.5. External Validation Performance

**Figure 4:**
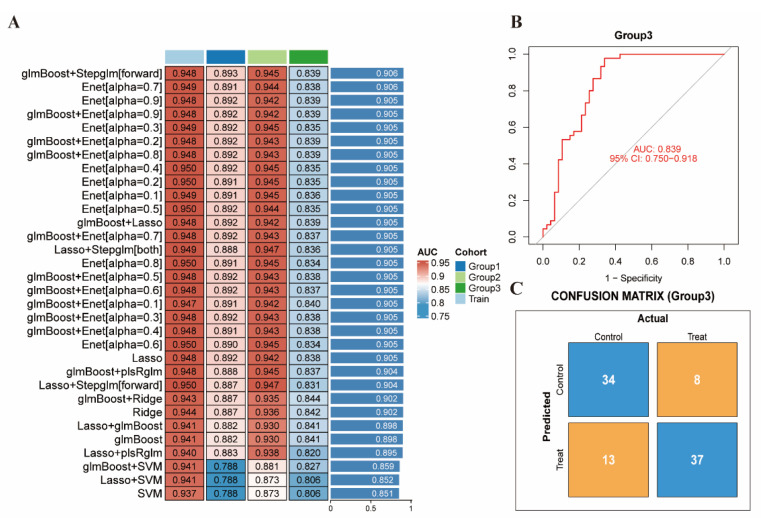
Results of external validation test. (**A**) AUC comparison between different machine learning combinations in external validation. (**B**) ROC curve plot in validation. (**C**) Confusion matrix plot in external validation.

To further evaluate the generalization ability of the model, we conducted independent testing on an external cohort (*N* = 92) from Chongqing People’s Hospital. The baseline characteristics of ARHL patients and normal-hearing individuals in this cohort were generally consistent with those in the training set (Table 2). The AUC rankings of various machine learning methods after incorporating external validation are shown in Figure 4A. Consistent with our hypothesis, glmBoost+Stepglm[forward] demonstrated the most stable overall performance and achieved the highest AUC across the validation sets. The model demonstrated good discriminative ability in this external cohort, with an area under the ROC curve (AUC) of 0.839 (95% CI: 0.750–0.918) (Figure 4B). The confusion matrix showed (Figure 4C) that the model achieved an overall accuracy of 77.2% (71/92), with a sensitivity of 74% (37/50) and a specificity of 80.95% (34/42). In the external validation set, sensitivity was 82.2% (37/45), precision was 74% (37/50), F1 score was 77.9%, and specificity was 72.3% (34/47). This indicates that the model maintains good predictive stability and generalization performance on completely independent clinical data. Notably, the relatively modest sample size of this external cohort should be considered when interpreting these results.

### 3.6. External Validation in an Age-Matched Elderly Subgroup

**Figure 5:**
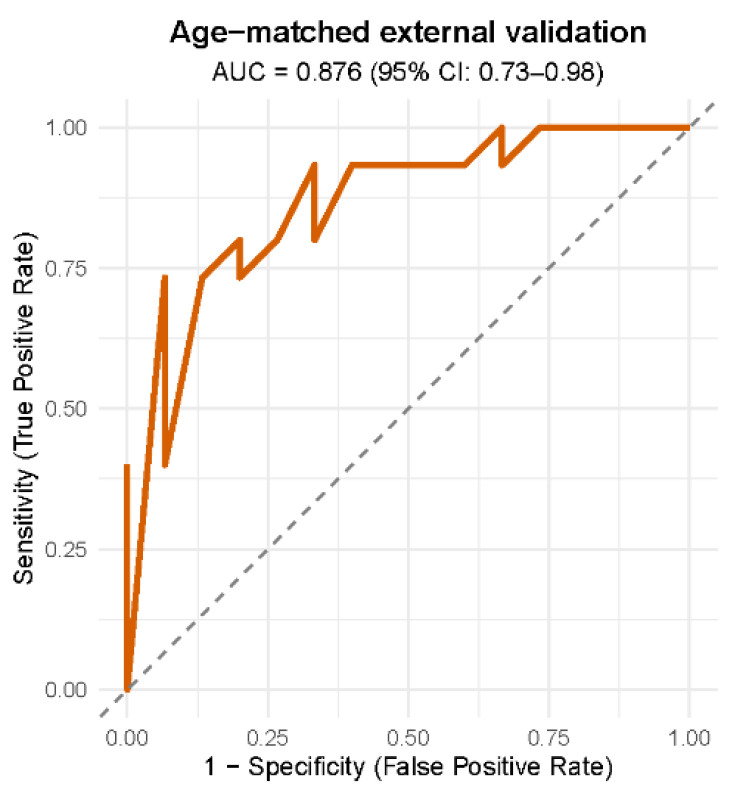
Receiver operating characteristic (ROC) curve of the 9-biomarker model in the age-matched elderly subgroup (1:1 matching, *n* = 30, age balanced).

To evaluate whether the model’s predictive performance is driven solely by chronological age, we performed an additional analysis using the external validation cohort (Chongqing dataset). We restricted the analysis to participants aged ≥60 years and conducted 1:1 nearest-neighbor age matching with a caliper of 2 years. This resulted in 15 ARHL patients and 15 normal-hearing controls with closely balanced age (mean age 70.1 ± 5.2 years vs. 69.9 ± 5.4 years, *p* = 0.85). In this age-matched elderly subgroup, the nine-biomarker model yielded an AUC of 0.876 (95% CI: 0.73–0.98) (Figure 5), indicating that the model retains strong discriminative ability even when chronological age is completely balanced between groups. The corresponding ROC curve is shown in Figure 5. This age-balanced validation demonstrates that the model’s predictive performance is not driven solely by chronological age, as the biomarker signature maintains high discriminative power even when age is strictly matched between groups.

### 3.7. SHAP Interpretability Analysis

**Figure 6:**
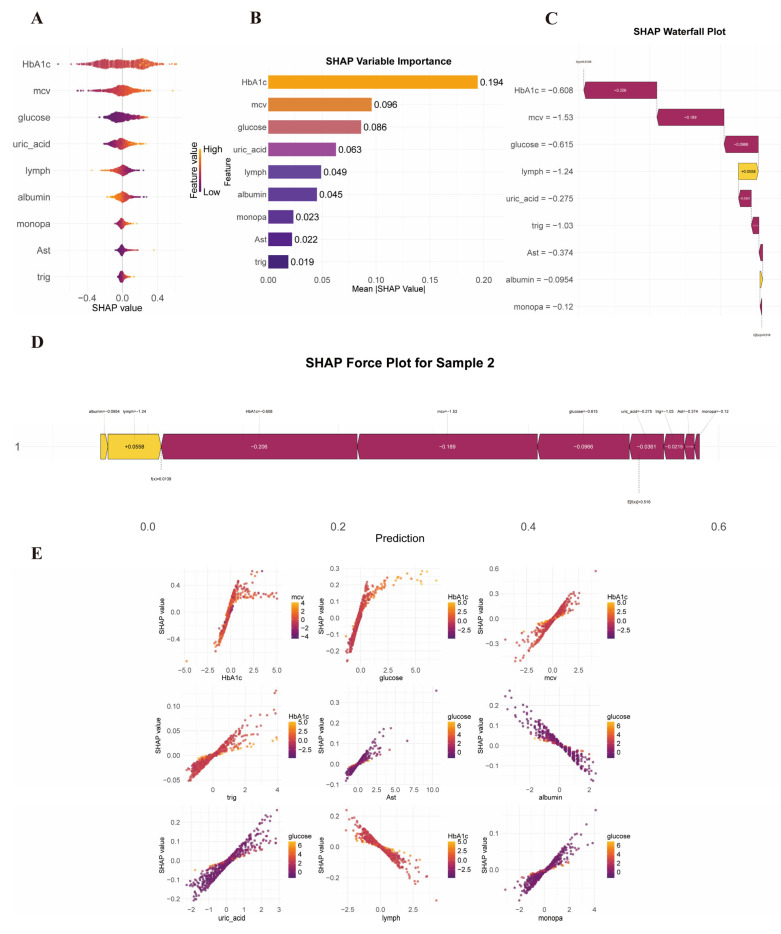
SHAPbased interpretability analysis of the prediction model. (**A**,**B**) Global feature importance plots. (**C**) Force plot of individual sample prediction. (**D**) Single sample baseline contribution chart. (**E**) Feature interaction scatter plot.

To elucidate the decision-making basis of the predictive model, we employed the SHAP method for interpretability analysis (Figure 6). Global analysis revealed (Figure 6A,B) that glycated hemoglobin (HbA1c), mean corpuscular volume (MCV), and blood glucose levels were the most significant predictive factors. To further understand the model’s decision logic at the individual level, we presented an analysis of a typical sample: the waterfall plot (Figure 6C) and force plot (Figure 6D) clearly demonstrated from different perspectives that the sample’s lower HbA1c and MCV values were the primary factors driving the model’s low-risk prediction. To gain deeper insights into how the synergistic effects of biomarkers influence ARHL risk prediction, we generated SHAP dependence scatter plots (Figure 6E) to quantify the conditional dependencies among various features in ARHL risk. In each subplot, the horizontal axis represents the actual measured values of the main feature, while the vertical axis indicates the SHAP contribution value of that feature to individual predictions. The color gradient of the points represents the values of the interacting feature (blue: low value; red: high value). The results revealed significant conditional dependencies between MCV and blood glucose/HbA1c features, as well as between MCV and triglycerides. Dependencies were also observed between triglycerides and monocyte count, between AST and blood glucose/albumin, between uric acid and albumin, and between lymphocyte count and HbA1c. These analyses not only enhanced the model’s transparency but also provided data-driven insights into the potential multifactorial interaction mechanisms underlying age-related hearing loss. We emphasize that all SHAP-based interpretations are associative and derived from the training data distribution; they do not imply causation.

### 3.8. Functional Demonstration of the Online Prediction Webpage

**Figure 7:**
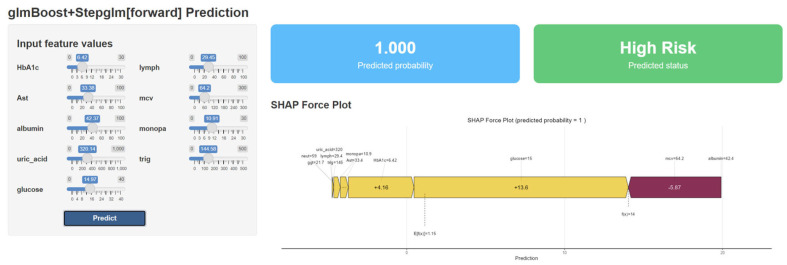
Web-based ARHL interactive prediction page.

To facilitate clinical translation and ease of use of the model, we developed a web-based interactive prediction interface (https://kj888.shinyapps.io/glmBoost-Stepglm/, accessed on 9 December 2024). Users can adjust biomarker values through an intuitive input panel, with the system performing real-time calculations to display predicted risk scores and interpretable visualization results. Figure 7 illustrates the webpage layout, comprising parameter input section, prediction output section, and SHAP decision force plot display area. A case example is presented: after entering a set of features, the webpage outputs a high-risk prediction (probability = 1.000) and generates corresponding SHAP decision force plots that clearly demonstrate the contribution direction and magnitude of each feature to the prediction outcome. This web-based tool combines usability with interpretability and can serve as an auxiliary screening instrument for clinical practitioners.

## 4. Discussion

The key finding of this study is that the machine learning model integrating multiple routine blood biomarkers demonstrated significantly superior performance in predicting age-related hearing loss (ARHL) compared to any single indicator. This strongly supports the view that ARHL is not dominated by a single etiology, but rather represents a complex pathophysiological process involving multiple systems and factors. While traditional research has predominantly focused on localized aging of the auditory system [23], our data-driven model incorporates systemic metabolic, immune, and nutritional status into a unified predictive framework, suggesting that ARHL shares partial biological foundations with systemic aging and various age-related comorbidities. To date, no prior machine learning model has been specifically developed for ARHL prediction using routine blood biomarkers. Existing studies have primarily focused on genetic hearing loss [11], noise-induced hearing loss [24], using diverse feature types such as genetic variants, audiometric parameters, or demographic characteristics. These fundamental differences in target disease, feature sets, and prediction objectives preclude a direct quantitative comparison of model performance. Our study therefore fills an important gap by providing the first interpretable, blood-based predictive model specifically for ARHL. To provide a structured overview of the existing literature, we summarize the key characteristics of previous hearing-loss prediction models in Table 3. As shown in Table 3, our model is the first to specifically target ARHL using routine blood biomarkers with external validation. Direct quantitative comparisons with prior models are limited by fundamental differences in target disease, feature types, and validation strategies.

A notable concern regarding the training dataset is the substantial age difference between the ARHL and control groups. However, since our model does not include age as a predictor, and given that the age-matched external validation (Figure 5) yielded an AUC of 0.876, we are confident that the nine blood biomarkers capture ARHL-relevant pathophysiology beyond the mere effect of aging. This is further supported by the observation that no single biomarker alone achieved performance comparable to the ensemble model (Figure 3C).

This study not only demonstrated excellent performance in internal validation, but also achieved robust predictive performance (AUC = 0.839) in a completely independent external cohort from Chinese medical institutions, further confirming the model’s strong cross-population generalization capability. Although the external cohort had a relatively small sample size (*n* = 92) and was from a single center in China, the results still indicated the model’s applicability across different healthcare settings and populations. The performance showed a slight decline compared to internal validation (AUC decreased from 0.893 to 0.839), which may reflect biological heterogeneity across populations, differences in laboratory assays between NHANES and the local hospital, or the limited sample size. Nevertheless, the model maintained clinically useful discriminative ability, supporting its potential as an auxiliary screening tool in various clinical scenarios. Accordingly, we propose that this model be used as a community-based prescreening tool to identify individuals who may benefit from formal audiological evaluation, rather than as a diagnostic test for age-matched comparisons.

Through systematic analysis using the SHAP framework, we have for the first time revealed in an interpretable manner the complex interaction patterns of multi-system biomarkers in ARHL risk assessment. Firstly, the global feature importance analysis consistently indicated that glycated hemoglobin (HbA1c) is the most significant feature influencing model predictions, with its higher SHAP values suggesting the close association between long-term glycemic control and the development of ARHL. This aligns with previous evidence linking diabetes and its complications to hearing loss [17,25,26]. It is noteworthy that mean corpuscular volume (MCV), as a hematological parameter, demonstrated secondary importance only to HbA1c in this model, suggesting potential alterations in erythrocyte morphology or volume may be associated with microcirculation or oxygen supply regulation in the inner ear. This offers correlational evidence for the hematological mechanism hypothesis of ARHL. Secondly, the interpretability of individual predictions further enhances the transparency of model decision-making. As illustrated by typical cases, lower HbA1c and MCV values collectively drove the model’s prediction toward lower risk. This indicates that in clinical practice, simultaneous attention to patients’ glycemic control and routine blood parameters may facilitate early identification of individuals with lower ARHL risk and provide basis for targeted health management. Most importantly, through SHAP dependence scatter plots, this study preliminarily identified conditional dependencies among multiple biomarkers, which may reflect the multifactorial pathogenic mechanisms of ARHL: the interactions between MCV and blood glucose, HbA1c, and triglycerides suggest a synergistic effect between abnormal glucose-lipid metabolism and changes in erythrocyte parameters [27,28,29], which may collectively influence the inner ear environment; the association between triglycerides and monocyte count indicates a certain correlation between lipid metabolism disorders and immune–inflammatory responses [30,31,32], which might explain the cross-talk between triglycerides and monocytes in ARHL progression; the mutual influence between AST (aspartate aminotransferase) and blood glucose as well as albumin [33,34] may contribute to age-related hearing impairment through liver function or the regulatory role of protein nutritional status in glucose metabolism; the association between uric acid and albumin, as well as between lymphocytes and HbA1c, reflects the complex relationships among various systemic systems, further supporting the notion that interactions among metabolism, immunity, and nutrition contribute to age-related diseases [35,36,37], which also represents one manifestation of the intricate pathophysiological processes underlying ARHL. The interaction patterns among these features extend beyond the explanatory scope of traditional single-factor analyses or linear models, demonstrating the advantages of machine learning models in capturing complex biological network relationships. They not only enhance the model’s credibility and clinical interpretability but also provide novel, data-driven insights into the multi-system, multi-mechanism pathogenic characteristics of ARHL.

Our study systematically compared over a hundred machine learning algorithms and subsequently constructed a glmBoost+Stepglm[forward] model. This model demonstrated not only high accuracy (AUC 0.893–0.948) and stable generalization capability in internal validation, but also maintained robust predictive performance (AUC 0.839) in an independent external clinical cohort, showcasing its potential for cross-population applications. More importantly, leveraging the SHAP framework, this research provided in-depth interpretation of the model’s decision-making logic, revealing the significance of key blood biomarkers such as HbA1c, MCV, and blood glucose, along with their interactive relationships. This approach bridges the predictive outcomes with potential multi-system interaction mechanisms involving metabolism, immunity, and hematopoiesis, thereby significantly enhancing the model’s interpretability and clinical acceptability. Furthermore, this study advanced the translation of models into clinical practice by developing an open-access interactive prediction web tool based on R Shiny. This tool enables real-time risk assessment and visual interpretation without requiring programming expertise, providing a convenient approach for early screening while laying the foundation for future integration into electronic health systems.

Nevertheless, this study has certain limitations. The training data primarily originated from the US NHANES cross-sectional database. Although externally validated, its population representativeness remains constrained by geographical coverage and relatively small sample size, and the cross-sectional design cannot establish causal relationships. The retrospective study design may also introduce residual confounding. Additionally, we acknowledge the inherent age imbalance between groups in the training dataset (median 72 vs. 28 years), which is largely attributable to the case–control design and the limited availability of elderly normal-hearing participants in NHANES. Furthermore, the age-matched external subgroup, while providing direct evidence against the ‘age-only’ hypothesis, had a relatively small sample size (*n* = 30). Despite these limitations, our age-balanced validation (AUC = 0.876) supports the ARHL-specific nature of the biomarker signature, indicating that the model captures pathophysiological signals beyond chronological age. Nonetheless, larger prospective cohorts with better age distribution are needed to further validate this signature. While our single-feature performance comparisons (Figure 3C) and differential expression analyses (Figure 3B) collectively support the predictive value of each of the nine biomarkers, we acknowledge that a formal ablation analysis—sequentially removing each feature and re-training the model—was not performed in the current study and represents a valuable direction for future work to further quantify each biomarker’s marginal contribution. Regarding feature coverage, the model only incorporated conventional blood indicators and did not include genetic information, detailed environmental exposure history, or imaging data, which may affect the comprehensiveness of predictions. Although SHAP analysis revealed interaction patterns among features, its interpretation still relies on the distribution of training data and has not been further validated in prospective cohorts or experimental studies. The external validation cohort, while providing valuable cross-population evidence, was relatively small (*n* = 92) and derived from a single center. This limits the generalizability of our findings. Future multi-center, large-scale prospective studies are needed to further confirm the model’s robustness across diverse ethnic groups and clinical settings. We also acknowledge that an additional sensitivity analysis restricting the training data to participants aged ≥50 years was not performed in the current study. Such an analysis would substantially reduce the training sample size given the limited availability of elderly normal-hearing participants in NHANES, and would deviate from the established model training pipeline. We therefore consider this a valuable direction for future research.

In subsequent research, we will further validate and optimize this model in multi-regional, multi-center prospective cohorts and explore its community application scenarios in combination with simple hearing screening. Meanwhile, incorporating multidimensional data such as genetic information and longitudinal dynamic monitoring indicators is expected to establish a more robust risk prediction system. Furthermore, it is necessary to conduct experimental studies to thoroughly validate the specific biological mechanisms of the metabolism–immune interaction axis in the development of ARHL as suggested by this research, thereby providing new targets for early intervention.

## 5. Conclusions

This study successfully developed and validated a high-accuracy, interpretable machine learning model based on routine blood biomarkers for community-based screening and risk stratification of age-related hearing loss (ARHL). The model demonstrated robust predictive performance across internal and external validations, including an age-matched elderly subgroup where it achieved an AUC of 0.876. An interactive web tool has been developed to facilitate real-time risk assessment. We emphasize that this tool is intended as a community-based prescreening aid for risk stratification (e.g., community health screenings) and should not be used as a standalone diagnostic test, particularly not for direct diagnostic comparisons between age-matched individuals. Nevertheless, larger multi-center prospective studies are warranted to further establish the model’s generalizability across diverse populations and clinical settings. This research offers a practical tool for early risk identification and provides data-driven insights into the systemic pathogenesis of ARHL.

## Figures and Tables

**Table 1:** Characteristics of the cohort participants in primary analysis.

Characteristics	ARHL (Median [Q1, Q3])	Control (Median [Q1, Q3])	*p*-Value
PTA (dB)	38.75 [33.75, 46.25]	2.50 [1.25, 3.75]	<0.001
age (years)	72 [65, 80]	28 [25, 32]	<0.001
HbA1c (%)	5.70 [5.40, 6.20]	5.20 [5.00, 5.40]	<0.001
Alt (U/L)	20.00 [16.00, 26.00]	18.00 [15.00, 25.00]	0.0208
Ast (U/L)	24.00 [21.00, 27.00]	21.00 [18.00, 25.00]	<0.001
total protein (g/L)	72.00 [68.00, 75.00]	73.00 [70.00, 76.00]	0.0316
albumin (g/L)	42.00 [40.00, 44.00]	44.00 [41.00, 46.00]	<0.001
ggt (U/L)	20.00 [15.00, 28.00]	15.00 [12.00, 20.50]	<0.001
uric acid (umol/L)	339.00 [291.50, 401.50]	291.50 [237.90, 339.00]	<0.001
phosphorus (mmol/L)	1.16 [1.07, 1.29]	1.19 [1.07, 1.29]	0.6825
total calcium (mmol/L)	2.35 [2.30, 2.40]	2.33 [2.27, 2.38]	<0.001
totchol (mmol/L)	5.07 [4.32, 5.71]	4.60 [4.00, 5.29]	<0.001
alp (U/L)	4.23 [4.06, 4.45]	4.13 [3.93, 4.29]	<0.001
bun (mmol/L)	2.83 [2.64, 3.04]	2.56 [2.30, 2.71]	<0.001
creat (umol/L)	4.42 [4.26, 4.59]	4.22 [4.01, 4.45]	<0.001
glucose (mmol/L)	5.49 [5.05, 6.38]	4.83 [4.55, 5.16]	<0.001
ttbl (umol/L)	0.70 [0.50, 0.90]	0.60 [0.40, 0.70]	<0.001
basopa (%)	0.70 [0.40, 0.90]	0.60 [0.40, 0.80]	0.0247
lymph (%)	27.70 [22.50, 31.70]	31.10 [25.45, 36.60]	<0.001
mcv (fL)	91.50 [88.30, 95.10]	89.50 [86.10, 92.70]	<0.001
monopa (%)	8.10 [6.80, 9.75]	7.40 [6.20, 8.90]	<0.001
neut (%)	60.40 [54.45, 65.70]	58.00 [52.00, 64.20]	0.0073
rbc (×10^12^/L)	4.67 [4.28, 4.99]	4.72 [4.35, 5.10]	0.1334
ldl (mg/dL)	111.00 [85.50, 137.50]	102.00 [84.50, 125.50]	0.0288
trig (mg/dL)	122.00 [86.50, 165.00]	83.00 [55.50, 129.50]	<0.001

**Table 2:** Characteristics of the External validation cohort participants in primary analysis.

Characteristics	ARHL (Median [Q1, Q3])	Control (Median [Q1, Q3])	*p*-Value
PTA (dB)	43.75 [32.50, 57.50]	7.50 [4.38, 18.12]	<0.001
age (years)	75 [68, 81]	46 [34, 60]	<0.001
HbA1c (%)	6.70 [5.90, 7.20]	5.70 [5.30, 6.20]	<0.001
Ast (U/L)	21.00 [17.00, 24.00]	18.00 [15.50, 19.30]	0.002
albumin (g/L)	40.80 [36.40, 43.10]	39.00 [38.00, 41.80]	0.950
uric acid (umol/L)	340.60 [291.30, 384.80]	251.30 [229.00, 338.80]	<0.001
glucose (mmol/L)	6.06 [5.46, 7.69]	5.16 [4.83, 5.92]	<0.001
lymph (%)	23.80 [19.70, 28.90]	32.60 [29.25, 37.25]	<0.001
MCV (fL)	93.10 [89.20, 95.60]	89.20 [84.25, 91.40]	<0.001
trig (mg/dL)	118.59 [74.34, 147.79]	103.54 [74.06, 128.61]	0.421
monopa (%)	6.80 [5.60, 7.80]	6.60 [5.70, 8.20]	0.758

**Table 3:** Summary of state-of-the-art machine-learning models for hearing-loss prediction.

Study	Target Disease	Data Type	ML Method	AUC
Chen et al. (2024) [11]	Genetic HL (GJB2-related)	Genetic variants	RF	0.82
Jafari et al. (2025) [14]	Tinnitus & NIHL	Audiometric, demographic	Ensemble	0.90
Gathman et al. (2023) [12]	General HL	Demographics, subjective hearing	XGBoost	0.79
Lenatti et al. (2022) [13]	Speech-in-noise detected HL	Speech test features	SVM	0.76
Our study	ARHL	routine blood biomarkers	glmBoost +Stepglm	0.929

## Data Availability

Data in this manuscript were obtained from online databases (https://www.cdc.gov/nchs/nhanes/, accessed on 9 December 2024) and all the new data has been fully incorporated in the text. The R scripts and preprocessing pipeline are available as Supplementary Materials with this manuscript.

## Supplementary Materials

The following supporting information can be downloaded at: https://www.mdpi.com/article/10.3390/diagnostics16132025/s1, R code.

## Abbreviations

The following abbreviations are used in this manuscript:

ARHL	Age-related hearing loss
NHANES	National Health and Nutrition Examination Survey
PTA	Pure-tone average
AUC	Area under the curve
ROC	Receiver operating characteristic
SHAP	SHapley Additive exPlanations
HbA1c	Glycated hemoglobin
MCV	Mean corpuscular volume
CI	Confidence interval
CDC	Centers for Disease Control and Prevention
HDL-C	High-density lipoprotein cholesterol
LDL-C	Low-density lipoprotein cholesterol
HL	Hearing loss
NIHL	Noise-induced hearing loss
RF	Random forest
SVM	Support vector machine
XGBoost	Extreme gradient boosting
AST	Aspartate aminotransferase
