# Supplementary material for: Development and Validation of an Interpretable Machine Learning Model Based on Routine Blood Biomarkers: For Predicting Age-Related Hearing Loss
Source: Diagnostics (Basel). 2026 Jun 29;16(13):2025. doi: 10.3390/diagnostics16132025 (PMC13360200; doi:10.3390/diagnostics16132025)
Supplement: Supplementary file 1 [file diagnostics-16-02025-s001.zip › README.pdf]

```
# ARHL Predictive Model - Code Package
#
# Preprocessing steps:
#     1. Z-score standardization on training set features
#     2. Apply same transformation to validation sets
#
# Model training:
#     - glmBoost + Stepglm[forward]
#     - 10-fold cross-validation
#     - Hyperparameter tuning via grid search and Bayesian optimization
#
# Files included:
#     - data_preprocessing.R
#     - model_training.R
#     - model_validation.R
#     - SHAP_analysis.R
#     - final_model.rds
#
# Dependencies: R version 4.3.0, packages: glmboost, caret, SHAPforxgboost, etc.
```
